# A density map-based method for counting wheat ears

**DOI:** 10.3389/fpls.2024.1354428

**Published:** 2024-05-01

**Authors:** Guangwei Zhang, Zhichao Wang, Bo Liu, Limin Gu, Wenchao Zhen, Wei Yao

**Affiliations:** ^1^ College of Information Science and Technology, Hebei Agricultural University, Baoding, China; ^2^ Hebei Key Laboratory of Agricultural Big Data, Hebei Agricultural University, Baoding, China; ^3^ State Key Laboratory of North China Crop Improvement and Regulation, Baoding, China; ^4^ College of Agronomy, Hebei Agricultural University, Baoding, China; ^5^ Key Laboratory of North China Water-savinssg Agriculture, Ministry of Agriculture and Rural Affairs, Baoding, Hebei, China

**Keywords:** counting wheat ears, instance segmentation, density map, CBAM, GeM pooling

## Abstract

**Introduction:**

Field wheat ear counting is an important step in wheat yield estimation, and how to solve the problem of rapid and effective wheat ear counting in a field environment to ensure the stability of food supply and provide more reliable data support for agricultural management and policy making is a key concern in the current agricultural field.

**Methods:**

There are still some bottlenecks and challenges in solving the dense wheat counting problem with the currently available methods. To address these issues, we propose a new method based on the YOLACT framework that aims to improve the accuracy and efficiency of dense wheat counting. Replacing the pooling layer in the CBAM module with a GeM pooling layer, and then introducing the density map into the FPN, these improvements together make our method better able to cope with the challenges in dense scenarios.

**Results:**

Experiments show our model improves wheat ear counting performance in complex backgrounds. The improved attention mechanism reduces the RMSE from 1.75 to 1.57. Based on the improved CBAM, the R2 increases from 0.9615 to 0.9798 through pixel-level density estimation, the density map mechanism accurately discerns overlapping count targets, which can provide more granular information.

**Discussion:**

The findings demonstrate the practical potential of our framework for intelligent agriculture applications.

## 1 Introduction

China is a populous country, a chronic shortage of food, and a supply that is significantly smaller than the demand is the main feature of the food market in China at this stage. As one of China’s major food crops, wheat plays an important role in ensuring food supply. In order to ensure national food security and cope with the fluctuation of wheat consumption on the market, it is necessary to predict the production of wheat in advance and formulate corresponding measures and policies in order to keep the market stable and meet people’s daily needs. Therefore, accurate estimation of wheat production is important to meet the growing demand for food, promote sustainable development of agriculture, and maintain national food security. Field wheat ear counting is an important step in wheat yield estimation, and how to solve the problem of rapid and effective wheat ear counting in a field environment to ensure the stability of food supply and provide more reliable data support for agricultural management and policy making is a key concern in the current agricultural field.

In the early days, traditional manual measurement methods required manual counting along the wheat field, which was slow, susceptible to human bias, and inefficient. Some methods are available to evaluate crops in an experimental setting (Bi et al., 2010; Kun et al., 2011; Qiongyan et al., 2017), but this ignores the effect of the complex context of the natural environment on the effectiveness of the evaluation. Image processing techniques have been used for wheat ears recognition, but the methods mainly focus on texture features (Anubha Pearline et al., 2019; Pérez-Rodríguez and Gomez-García, 2019), color segmentation, morphology extraction and other feature extraction methods. Feature extraction relies on manually designed feature extractors, which are suitable for application to some specific scenarios, so their generalization ability is poory.

With the rise of machine learning, in order to improve the accuracy and robustness of recognition, researchers use classification techniques such as support vector machines for wheat counting. (Zhou et al., 2023) used a feature selection algorithm based on the principle of compact separation (FS-CS) to filter spectral and textural features extracted from time-series UAV images, and a multilevel correlation vector machine (mRVM) to classify the main phenological stages, which was tested in wheat fields during two experimental seasons, and the experimental results showed that the best estimation was generated with the use of FS-CS and mRVM when the number of optimal features was small. (Bao et al., 2023) in order to improve the accuracy of wheat yield estimation, a wheat ears count method based on frequency domain decomposition is proposed. A combination of multi-scale support value filter (MSVF) and improved sampling contour transform (ISCT) is used to decompose the wheat ears image in the frequency domain, reduce the interference of irrelevant information, and generate a subband image with richer information components in the ear feature information, and experiments show that compared with the traditional algorithm based on the spatial domain, the method significantly improves the accuracy of the wheat ears number, and provides the field of accurate agricultural yield estimation provide guidance and application. All of the above methods use data labeling, but it is labor intensive, so the researchers again proposed unsupervised segmentation of the wheat ear. For example (Xu et al., 2020) used k-mean clustering technique to automatically segment the images of wheat ears counts collected by handheld devices for fast and accurate wheat ears counts, and the recognition rate of wheat reached up to 98.5%. Machine learning is able to learn target features from given data to achieve better recognition, so the accuracy of target feature selection determines the effectiveness of this type of method, but it needs to be determined by the researcher to determine the target features, which is subjective.

In recent years, deep learning has developed rapidly and has proven to have significant advantages in the field of machine vision, especially the application of convolutional neural network (CNN) (Gu et al., 2018) in image analysis is starting to become mainstream, such as image segmentation and target recognition (Jo et al., 2019; Zhu et al., 2019). Deep learning techniques have achieved great success in image recognition, natural language processing, and time series analysis. In agriculture, these techniques can be applied for identifying diseases, predicting crop growth, optimizing agricultural production processes, etc., thus improving the efficiency and quality of agricultural production. Deep learning methods are gradually being used by most researchers to solve agricultural problems.

Nowadays, wheat ears detection algorithms have received extensive research and attention. One of them (Zhao et al., 2022) introduced a wheat ears detection algorithm based on the improved YOLOv4 algorithm. In order to enhance the receptive field, an additional spatial pyramid pool (SPP) block was added to YOLOv4 in the feature fusion part to extract multi-scale features. In order to make more full use of the underlying information and to solve the problem of poor detection of wheat ears, (Sun et al., 2022a) proposes a wheat ears counting method based on the augmented feature pyramid network of convolutional neural network, which utilizes augmented feature pyramid network (AugFPN) for raw information to perform Adaptive convergence makes full use of the underlying information to solve the problem of poor detection of wheat ears. Experiments on test set images show that the method has an average error rate of 3.7% and an AP of 95.17%, which is significantly better than other state-of-the-art methods. (Li et al., 2022) an image wheat counting method based on Faster R-CNN algorithm and applied to genetic research, realizes the automatic counting of wheat images and applies the method to the task of wheat counting in genetic research. The experimental results show that the method can effectively perform wheat ears counting and has some application value in genetic research. (Shen et al., 2023b) proposes an enhanced YOLOv5 algorithm incorporating separable convolution and attention mechanisms to cope with the challenges posed by different wheat varieties, planting densities, lighting conditions and complex backgrounds. Compared with YOLOv5, the improved algorithm achieves 4.2% improvement in mAP and 1.3% improvement in FPS, and outperforms other YOLO series algorithms and mainstream detection algorithms in processing high-resolution images. These methods achieve accurate counting of wheat ears through deep learning techniques, which improves the accuracy and robustness of wheat ear detection.

On the other hand, some researches have introduced the attention mechanism to improve the wheat detection algorithm. To address the problem of vanishing gradients during training (Li and Wu, 2022) adding quadruple downsampling in YOLOv5 to improve the detection effect of small targets and adding the CBAM attention mechanism in the backbone network to solve the gradient disappearance problem during the training process, and the results show that, compared with the other methods, the model has a mAP of 94.3%, an accuracy of 88.5% and a recall of 98.1%, which is an obvious improvement. For rapid detection of wheat (Zhaosheng et al., 2022) proposes a fast method for wheat ears orthophoto detection based on YOLOX algorithm for UAV aerial photography, which adds a channel attention mechanism in the backbone and necks a Bidirectional Convolutional Block Attention Module (BiFPN) structure, which uses learnable weights to learn the importance of different input features. Experimental results show that the method exhibits good accuracy and efficiency in the task of wheat sheaf detection in aerial wheat field images, providing a fast and feasible solution for wheat sheaf detection in aerial UAV wheat fields. (Sun et al., 2022b) introduced a high-performance wheat ears detection method based on WDN (one-stage detection network), by adding an attention module and a feature fusion module to the structural backbone network, the authors realized a high-precision detection of wheat ears, and the mAP metrics of the WDN model outperformed the other models, reaching 90.3%. (Dong et al., 2022) utilized lightweight backbone network with asymmetric convolution for feature extraction, after which SPSA attention was used to select the focusing location and generate more discriminative feature representations. The method introduces both spatial and channel dimensions to the polarized self-attention and employ disordered cells to effectively combine these two attention mechanisms. Experimental results on Global Wheat Detection Data (GWHD) show that the proposed methods have better detection performance compared to other state-of-the-art methods. By introducing the attention mechanism, these methods are able to better focus on important regions of the wheat ears let, improving detection accuracy, speed and adaptability. Therefore, these improved methods provide a useful exploration and development direction for the research and application of wheat ears detection algorithms.

Some researchers have begun to use deep learning architectures to improve the accuracy of segmentation-based wheat counting, (Zaji et al., 2022) proposes a method for wheat ears localization and counting using a hybrid UNet architecture. The method makes full use of the encoder-decoder architecture and jump connections to achieve accurate ear localization and counting by performing effective feature extraction and contextual information transfer to wheat ears. (Ma et al., 2020) proposes a semantic segmentation method for accurate segmentation of winter wheat, and its method uses the encoder-decoder structure and extended convolution, with a segmentation quality of 0.7743, an F1 score of 87.25%, and a structural similarity index of 0.8773, which is better than the comparison method. (Zhang et al., 2022) proposes the automatic dense wheat ears segmentation method named Wheat-Net, which adopts an optimized hybrid cascade task model, and achieves a high-density and accurate segmentation of wheat ears through a multi-stage cascade structure and an attention mechanism, providing an automated solution for wheat ears segmentation. These studies have made significant progress toward accurate and efficient wheat ears segmentation, paving the way for improved agricultural research and crop yield estimation. To effectively deal with adhesion, (Shen et al., 2023a) proposes an improved Mask R-CNN-based algorithm for unsound wheat kernel segmentation, which achieves faster and more accurate unsound kernel recognition by means of a bottom-up feature pyramid network and by adding an Attention Mechanism (AM) module between the feature extraction network and the pyramid network, and at the same time effectively handles the sticking problem to achieve an accuracy of 86% and a recall of 91%. The inference time of this model on the test set is 7.83 seconds, which is significantly better than other segmentation models and provides an important foundation for wheat grading. Deep learning does not rely on manual feature extraction and has a very strong learning capability that improves the accuracy and robustness of wheat counting.

However, there are still some bottlenecks and challenges in solving the dense wheat counting problem with the currently available methods. The dense target counting problem has many difficulties. In dense scenes, targets often occlude each other, which may result in some of them not being fully detected or accurately counted. In addition, the shapes of dense targets are often irregular, which increases the complexity of detection and counting. Conventional detection and counting methods may perform poorly when facing these situations. Complex background environments are also a challenge, as some background elements may be incorrectly recognized as targets, leading to false detections. Therefore, dense target counting methods need to be highly adaptable and able to cope with scenarios of varying densities and complexities in order to improve the accuracy and robustness of counting.

To address these issues, we propose a new method based on the YOLACT (Bolya et al., 2019) framework that aims to improve the accuracy and efficiency of dense wheat counting. The pooling layer in the CBAM (Woo et al., 2018) module is replaced with the GeM pooling layer so that the model learns the features better and retains the spatial information of the features better. Then the density map is introduced into the Feature Pyramid Networks(FPN), and the feature maps of different scales output by the FPN are converted into density maps of the same dimensions and combined with the Protonet head to further improve the accuracy of the detection results. The Protonet branch is linearly combined with additional prediction head networks to generate the final mask.

Our proposed YOLACT-based method has significant potential for the dense target counting problem, and these improvements work together to make our method better able to cope with the challenges in dense scenarios and improve the effectiveness and reliability of wheat counting. In the next sections, we describe our method in detail as well as the experimental results.

In summary, our contributions in this paper are as follows:

1) We introduce the CBAM attention module in the backbone network of YOLACT. This module helps increase the model’s attention to features, thus improving the performance of dense target counting. We replace the pooling layer in the CBAM module with the GeM pooling layer, which provides more flexibility and allows better capture of target features, especially in dense scenes. By adjusting the hyperparameters of GeM pooling, we enable adaptive pooling according to different densities of the target scene, improving the feature representation.
2) To cope with the dense target counting problem, we introduce the density map mechanism. By applying loss to the feature map and performing back propagation, our method better understands the target distribution, especially in dense scenes. This mechanism reduces leakage and false detection, improving the accuracy of counting.
3) Experiments on the GWHD, WEDD, and ESD datasets validate our method’s effectiveness in dense wheat ear counting, achieving mAP scores of 64.41%, 61.62%, and 65.31%, respectively. Our approach enables accurate target capture in dense scenes and significantly improves counting performance compared to existing methods.

## 2 Materials and methods

### 2.1 Materials

#### 2.1.1 Data collection

In this study, three datasets were used to validate the performance of the model, including two public datasets and one self-built dataset named Experimental Station Dataset(ESD). The Global Wheat Detection Dataset (GWHD) (David et al., 2021), containing 6500 RGB images (1024 × 1024 pixels) and 275187 wheat heads from 16 institutions distributed in 12 countries, including Europe (France, UK, Switzerland), North America (Canada), Oceania (Australia) and Asia (Japan, China). The images collected vary widely, including different varieties, different collection methods and different growing conditions. Therefore the wheat samples in this dataset are diverse and typical. See **Table 1** for specific information. Another public dataset is the Wheat Ears Density Dataset(WEDD) (Madec et al., 2019), containing 240 RGB images, was collected from Gréoux-les-Bains (France, 43.7°N, 5.8°E) using a 6000 x 4000 pixel Sony ILCE-6000 digital camera, taken from the direction of the lowest point view at 2.9 m above the ground. Ground sampling distances ranged between 0.010 - 0.016 cm/pixel, and the area of individual images ranged between 0.25 m² and 0.56 m².

**Table 1:** Dataset information.

Sub- dataset	Country	YEAR	Lat	Long	Camera	Distance to ground (m)
UTokyo_1	Japan	2018	36.0 N	140.0 E	2*Canon G9 X mark II	1.8
UTokyo_2	Japan	2016	42.8 N	143.0 E	2*Olympus 850Sony DSC-HX90V	1.7
Arvalis_1	France	2017	43.7 N	5.8 E	Sony alpha ILCE-6000	2.9
Arvalis_2	France	2019	43.7 N	5.8 E	Sony RX0	1.8
INRAE_1	France	2019	43.5 N	1.5 E	Sony RX0	1.8
USask_1	Canada	2019	52.1 N	106 W	FLIR Chameleon3 USB3	2
RRes_1	UK	2016	51.8 N	0.36 W	Prosilica GT 3300 Allied Vision	2.2
ETHZ_1	Switzerland	2018	47.4 N	8.6 E	Canon EOS 5D mark II	3
NAU_1	China	2018	31.6 N	119.4 E	Sony RX0	2
UQ_1	Australia	2016	27.5 S	152.3 E	Canon 550D	2
WEDD	France	2017	43.7 N	5.8 E	Sony ILCE-6000	2.9
ESD	China	2022	37.4 N	115.1 E	Redmi K40 Pro	1

To verify the generalization ability of the wheat counting model, we also acquired wheat images in the field. The acquisition site was Xinji Experimental Station (ES,37°4’N, 115°1’E) of Hebei Agricultural University, with a field trial area of 0.1ha, and a two-factor split-zone test set up for irrigation and nitrogen application. The irrigation standard was the crop water requirement (CWR) calculated based on the Cropwat model, with normal irrigation (100% CWR, denoted by W100), mild water stress (The irrigation rate and N application rate were orthogonal to each other), and each plot was sampled three times and the results were averaged. During shooting, the equipment was positioned perpendicular to the ground at a 90-degree angle, approximately one meter above the ground. Images with obvious defects or blurriness were excluded to ensure the quality of the training data. The original image resolution was 4624 × 3472 pixels, which was then uniformly cropped to 1024×1024 pixels. In the end, 216 high-quality images of wheat ears were obtained.

**Figure 1:**
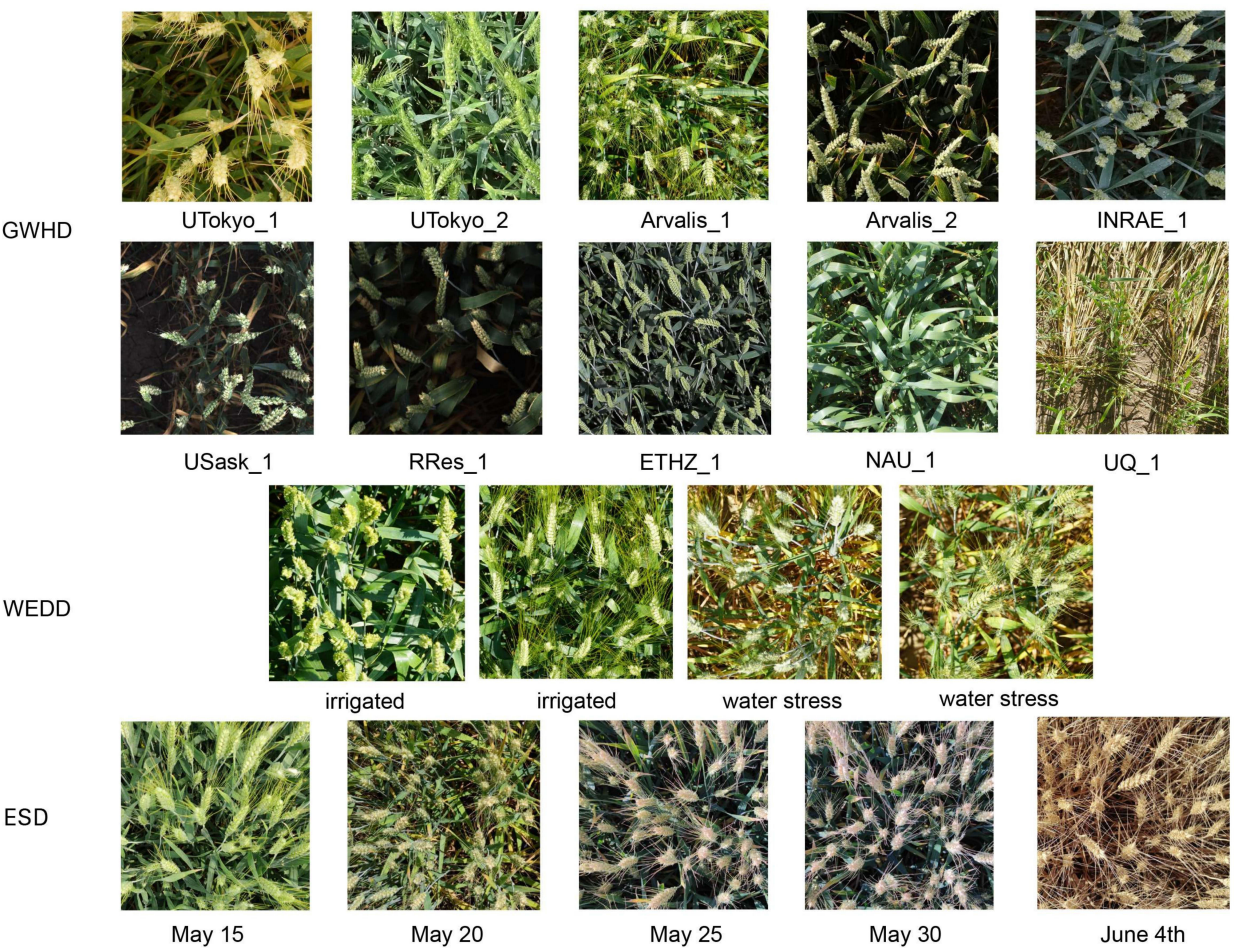
GWHD, WEDD, and sample views of our collected datasets.

We selected 1000 images from the GWHD dataset, 230 images from the WEDD dataset, and 216 images from the ESD, processed them to the same resolution of 1024 x 1024, and divided the processed dataset into training, validation, and test sets in an 8:1:1 ratio, ensuring a balanced and representative distribution for each set. A sample dataset is shown in **Figure 1**.

#### 2.1.2 Data annotation

In this paper, we use labelme (https://github.com/wkentaro/labelme) annotation software to annotate the dataset. The outline of each wheat ears is marked into a polygon by manual annotation, and the outlines of all wheat ears in each image are marked as their groundtruth labels. After that, the corresponding JSON files were generated, which included information such as the location and species label of each instance. After processing the dataset is stored in the format of COCO dataset.

#### 2.1.3 Generation of density maps

The feature maps of different scales output by Feature Pyramid Networks (FPN) (Lin et al., 2017) are converted into density maps of the same scale. The process of generating a density map usually consists of the following steps: first, for each target, the (x,y) coordinates of the center of the target are obtained by obtaining the annotation information of the json file to determine its precise location; then, for each target, a Gaussian distribution centered on the target location is created on the density map, and these Gaussian distributions are superimposed on top of each other, and this superposition process maps the information about the distribution of the successive targets onto the density map. Map resulting in a density map used to more accurately characterize the distribution of targets in the image, with the value of each pixel indicating the density estimate of the presence of a target in the vicinity of that location and the generation formula is as shown in Equation 1:


(1) $$D\left(a,b\right)=\sum_{i=1}^{N}\frac{1}{2\pi \sigma^{2}}\exp\;\left(-\frac{{\left(a-a_{i}\right)}^{2}+{\left(b-b_{i}\right)}^{2}}{2\sigma^{2}}\right)$$

where *N* is the number of targets, (*a_i_ ,b_i_*) is the central coordinate of the *i* target, and *σ* is the standard deviation of the Gaussian kernel function, which is used to control the ambiguity of the density map. The meaning of the formula is that on each pixel position (*a, b*) of the feature map, the distance from the center position of all targets is calculated, and then the density value is calculated according to the Gaussian kernel function. Finally, the density value of all targets is added up to get the final density map *D*.

### 2.2 Methods

**Figure 2:**
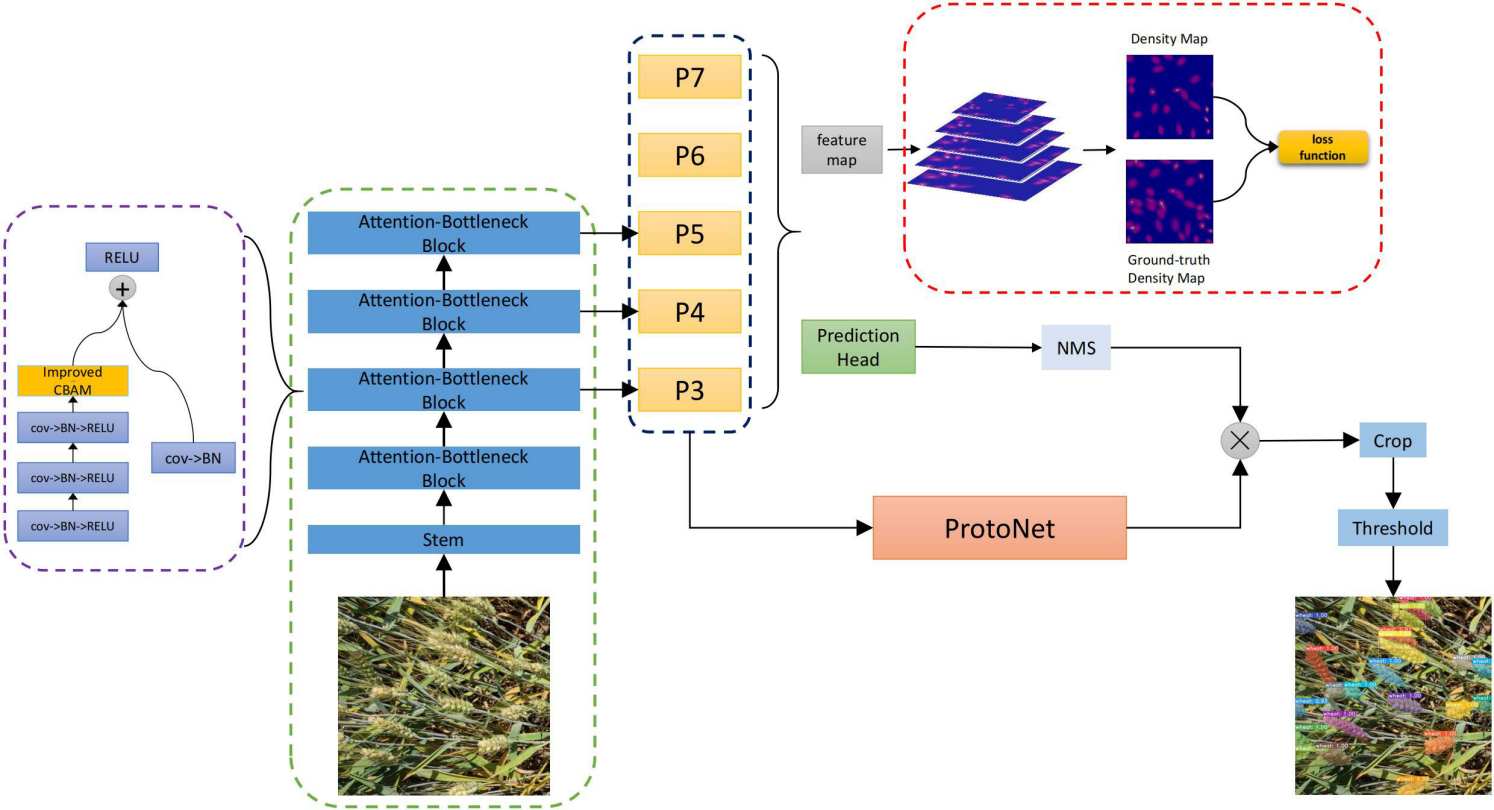
Improved network structure based on density.

In this paper, an improved YOLACT (Bolya et al., 2019) model is proposed, which aims to improve the accuracy and performance of object detection. We improve the backbone of YOLACT by introducing the CBAM (Woo et al., 2018) attention mechanism and the GeM (Radenović et al., 2018) pooling layer, and optimize the detection performance by introducing density map mechanism. The network structure proposed in this paper is shown in **Figure 2**.

#### 2.2.1 YOLACT

Specifically, the CBAM module includes two sub-modules: channel attention and spatial attention. The former weights features by learning the importance of channels, and the latter weights features by learning the spatial correlation of features. In this way, the model can learn the feature representation of the target more accurately. Meanwhile, we replace the pooling layer of YOLACT with the GeM pooling layer. Traditional pooling operations usually use fixed-size windows to downsample features, but this may lead to the loss of spatial information of features. In contrast, the GeM pooling layer can better preserve the spatial information of features by adaptively adjusting the size of the pooling window, thus improving the performance of the model. Secondly, the density map is introduced into FPN and combined with the detection head. The density map is generated based on the distribution of the target, which can provide more accurate target information. By fusing the density map with the detection head, we can better consider the distribution of the target in the target detection process, and further improve the accuracy of the detection results. The YOLACT is a one-stage real-time instance segmentation model that splits an instance into two parallel tasks. One branch is Protonet, which uses a full convolutional network to generate a series of prototype masks. The other branch is to add an additional prediction header network through the object detection branch, generating a set of prediction boxes, category information, and mask coefficients. Finally, the two branches are linearly combined to generate the final mask. The YOLACT network consists of three branches: backbone network, detection branch and mask branch. The YOLACT loss function is divided into three parts: classification loss ${ℒ}_{\text{cla}}$ , detection box regression loss ${ℒ}_{\text{detection}}$ and mask loss ${ℒ}_{\text{seg}}$ with the weights 1, 1.5, and 6.125 respectively. The formula is defined as shown in Equation 2:


(2) $${ℒ}_{\text{total}}=\lambda {ℒ}_{\text{detection}}+\lambda {ℒ}_{\text{seg}}+\lambda {ℒ}_{\text{cla}}$$

The object detection loss consists of two parts: the Objectness Score loss and the Bounding Box Localization loss. Given N prior boxes and C categories (including the background category), the object detection loss can be expressed as shown in Equation 3:


(3) $${ℒ}_{\text{detection}}={ℒ}_{\text{obj}}+{ℒ}_{\text{box}}$$

Objectness Score Loss function formula is as shown in Equation 4:


(4) $${ℒ}_{\text{obj}}=\sum_{i=1}^{N}\sum_{j=1}^{S^{2}}\text{binary}\_\text{cross}\_\text{entropy}\left(p_{i,j}^{\text{obj}},{\hat{p}}_{i,j}^{\text{obj}}\right)$$

where $p_{i,j}^{obj},$ the predicted target presence probability of the i-th prior box at the j-th position in the feature map, and ${\hat{p}}_{i,j}^{obj}$ is the corresponding real target presence label (1 means the target is present, 0 means the target is not present).

Bounding box localization loss function formula is as shown in Equation 5:


(5) $${ℒ}_{\text{box}}=\sum_{i-1}^{N}\sum_{j=1}^{S^{2}}\text{smooth}\_\text{L}1\_\text{loss}\left(t_{i,j}^{\text{box}},{\hat{t}}_{i,j}^{\text{box}}\right)$$

where, $t_{i,j}^{box}$ is the bounding box position prediction value of the i-th prior box at the j-th position in the feature map, and ${\hat{t}}_{i,j}^{box}$ is the corresponding true bounding box position label. The segmentation loss uses the cross-entropy loss function, which is used to measure the difference between the prediction of the network for each pixel segmentation mask and the true mask. Given P feature points and K classes, the segmentation loss can be expressed as shown in Equation 6:


(6) $${ℒ}_{\text{seg}}=\sum_{i=1}^{P}\sum_{k=1}^{K}\text{cross}\_\text{entropy}\left(m_{i,k}^{\text{seg}},{\hat{m}}_{i,k}^{\text{seg}}\right)$$

where, $m_{i,k}^{seg}$ is the segmentation mask prediction value of the k-th class corresponding to the i-th feature point, and ${\hat{m}}_{i,k}^{seg}$ is the corresponding true segmentation mask label.

The classification loss again uses the cross-entropy loss function, which is used to measure how different the network’s prediction for each target instance class is from the true class label. Given P feature points and K classes, the classification loss can be expressed as shown in Equation 7:


(7) $${ℒ}_{\text{cla}}=\sum_{i=1}^{P}\sum_{k=1}^{K}\text{cross}\_\text{entropy}\left(c_{i,k}^{\text{cls}},{\hat{c}}_{i,k}^{\text{cls}}\right)$$

where $c_{i,k}^{cls}$ is the category prediction value of the k-th category corresponding to the i-th feature point, and ${\hat{c}}_{i,k}^{cls}$ is the corresponding true category label.

#### 2.2.2 Attention module

**Figure 3:**
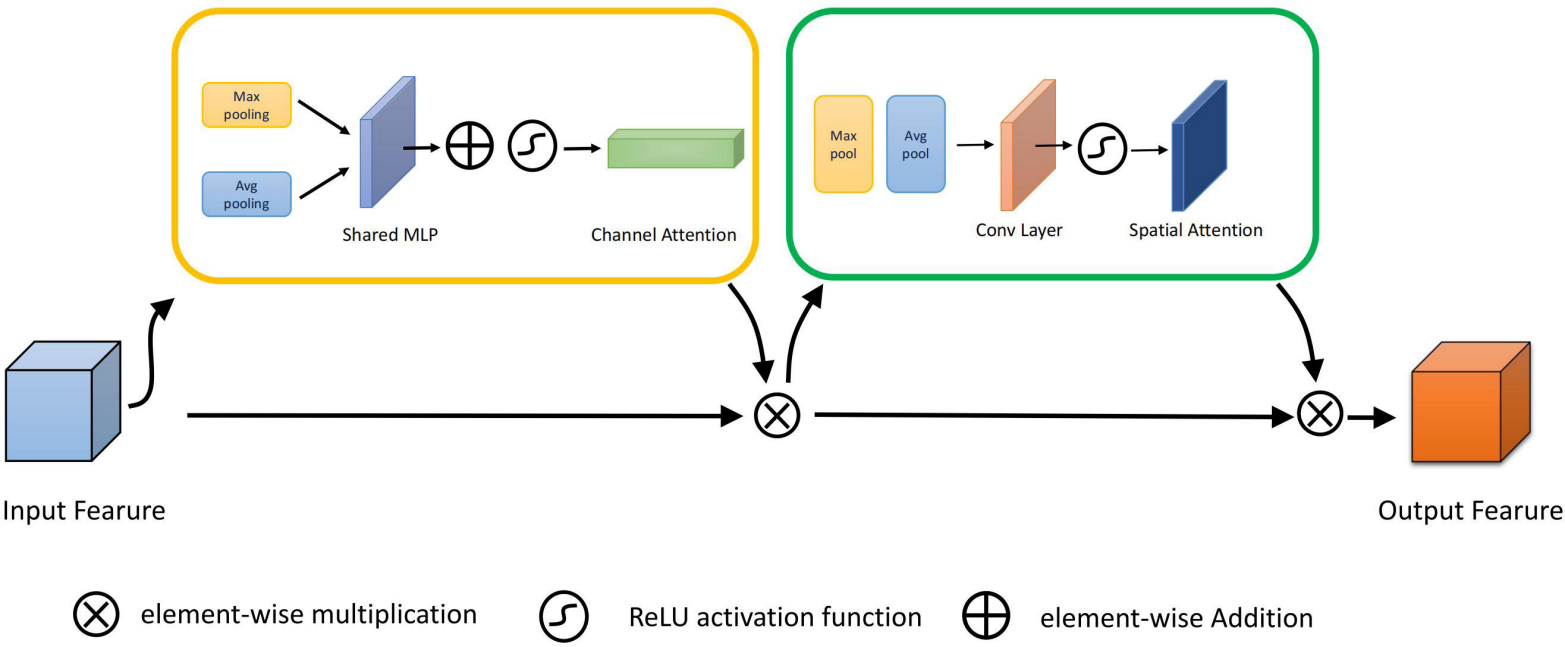
Original CBAM structure.

The accuracy of wheat detection is seriously affected by the overlap between wheat sheaves and the highly cluttered background. Therefore, we introduce the CBAM(Convolutional Block Attention Module) (Woo et al., 2018) attention module in backbone. The original CBAM module is shown in **Figure 3**. This module integrates spatial attention and channel attention mechanisms to selectively highlight information-rich features and suppress irrelevant or redundant information. The spatial attention mechanism allows CBAM to adaptively adjust the weights of the feature maps through spatial attention, enabling the network to focus on the salient regions of the wheat ears and ignore background noise. Meanwhile, the channel attention mechanism adaptively recalibrates the importance of different channels by capturing the dependencies between channels, enabling the network to emphasize information-rich channels and weaken irrelevant ones. By combining spatial and channel attention, CBAM can effectively capture local and global contextual information, thus improving feature representation and distinguishability.

#### 2.2.3 Improved attention module

**Figure 4:**
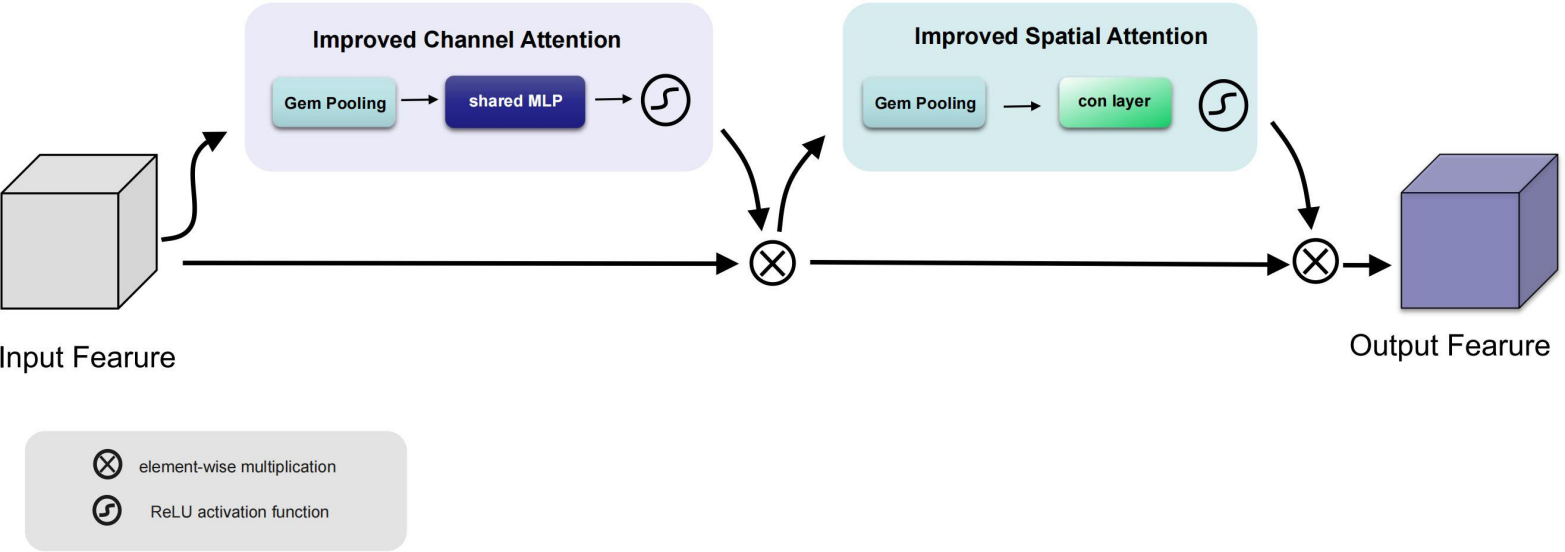
Improved CBAM structure.


In this paper, we propose a method to modify CBAM by replacing all pooling layers with GeM Pooling, a GeM-based pooling method that adaptively integrates spatial information, the specific improvement is shown in **Figure 4**. GeM pooling layers can be represented as shown in Equation 8:

(8) $$\text{GeM}\left(x\right)={\left(\frac{1}{H\cdot W}\sum_{i=1}^{H}\sum_{j=1}^{W}x_{i,j}^{p}\right)}^{\frac{1}{p}}$$

where GeM(x) is the output of the GeM pooling layer, x is the input feature map, and *H* and *W* are the height and width of the feature map. The *p* is the hyperparameter of GeM, which controls the power in summation. Specifically, when *p* = 1, the GeM Pooling layer degenerates into average pooling, that is, the values of all elements in the feature map are averaged. When *p* = ∞, the GeM pooling layer tends to select larger eigenvalues to highlight more significant features.

By using GeM pooling it is possible to capture finer information and preserve spatial detail than traditional pooling layers, making it possible to focus more precisely on spatial regions and better focus on the features of the target.

#### 2.2.4 Density map

We propose a new method for detecting and counting overlapping wheat ears using the concept of density estimation. We introduce a density-based method to estimate the local density of wheat ears in an image. By generating a density map, the location information of the target wheat ears can be observed more clearly, then the distribution of wheat ears is more accurately reflected. In order to integrate the density map into YOLACT, a new loss function is introduced as shown in Equation 9:


(9) $$\text{MSE}=\frac{1}{N}\sum_{i=1}^{N}{\left(d_{i}-gt_{i}\right)}^{2}$$

where *N* denotes the total number of pixels in the density map, $d_{i}$ denotes the predicted density pixel value, and $gt_{i}$ denotes the actual density pixel value, the value returned by the mean-square error(MSE) reflects the difference between the generated density map and the ground-truth, the smaller the MSE is, the closer the predicted density map is to the actual density map, and the bigger the MSE is, the farther away from the ground-truth.

Back propagation of the loss of the density map by the network drives the network to generate accurate density maps that are constantly close to the ground-truth. By co-optimizing the target detection and the loss of the density map, the network learns to use the density information and adaptively adjusts the detection strategy to show better performance when facing dense targets.

#### 2.2.5 Implementation details

All experiments in this paper are carried out under the PyTorch deep learning framework, and the system environment is 20.04.1-Ubuntu. The computer hardware configuration is 2080Ti 11GB graphics card, Intel(R) Core(TM) i9-9900X CPU @ 3.50GHz processor, pytorch version is 1.8.1, and python version is 3.8.8.

In this paper, we use stochastic gradient descent (SGD) algorithm to train the network, with the momentum coefficient set to 0.9, the initial learning rate to 0.001, the batch size to 8, and the input image to 1024×1024×3 (RGB). For the CBAM attention module, we set the initial values of P in the GeM pool layer to 11 and 19 for channel attention and spatial attention, respectively. Backbone is resnet-101, and the pre-trained weights from the COCO dataset are used to speed up the convergence.

#### 2.2.6 Evaluation metrics

To validate the accuracy and effectiveness of our proposed method, we use root mean square error (RMSE), Bias and coefficient of determination R² as evaluation metrics to measure the performance of model counting. The formulas are as shown in Equation 10:


(10) $$RMSE=\sqrt{\frac{1}{n}\sum_{i=1}^{n}{\left(y_{i}-{\hat{y}}_{i}\right)}^{2}}\;Bias=\frac{1}{n}\sum_{i=1}^{n}\left(y_{i}-{\hat{y}}_{i}\right)\;R^{2}=1-\frac{\sum_{i=1}^{n}{\left(y_{i}-{\hat{y}}_{i}\right)}^{2}}{\sum_{i=1}^{n}{\left(y_{i}-\bar{y}\right)}^{2}}$$

where $y_{i}$ denotes the ground-truth (the number of targets actually observed), ${\hat{y}}_{i}$ is the predicted value (the number of targets predicted by the model) denotes the ground-truth of the *i*-th observation, and *n* is the sample size. The RMSE measures the degree of difference between the actual observations and the predicted values. It is the result of squaring, averaging, and taking the square root of the prediction error, so it can be considered as the standard deviation of the prediction error. The *Bias* indicates that the forecast value deviates from the ground-truth on average, and a positive *Bias* indicates that the forecast value is high, while a negative *Bias* indicates that the forecast value is low. $\sum_{i=1}^{n}{\left(y_{i}-{\hat{y}}_{i}\right)}^{2}$ is the sum of squares of residuals (the sum of squares of residuals’ deviations), representing the difference between observed and predicted values. $\sum_{i=1}^{n}{\left(y_{i}-\bar{y}\right)}^{2}$ is the total sum of squares (total deviation squared), which represents the difference between the observed value and the ground-truth.

In addition this paper uses precision (P), recall (R) and average precision (AP), to evaluate the performance of the segmentation model as shown in Equation 11.


(11) $$R=\frac{TP}{TP+FN}\;P=\frac{TP}{TP+FP}\;AP=\frac{1}{\left|ℛ\right|}\sum_{r\in ℛ}AP_{r}\;mAP=\frac{1}{N}\sum_{i=1}^{N}AP_{i}$$

where *TP* (True Positive) denotes the number of samples whose model correctly predicts positive cases, *FN* (False Negative) denotes the number of samples whose model incorrectly predicts negative cases, and *FP* (False Positive) denotes the number of samples whose model incorrectly predicts positive cases. |ℛ| denotes the total number of true targets in the dataset. ℛ is the set of real targets, containing all real targets in the dataset. $AP_{r}$ is the Average Precision of a single target, indicating the accuracy of the predicted results for a single target. It is a measure of the prediction quality of a target by calculating the area under the precision-recall curve of the target. *N* is the number of samples, which indicates the total number of samples in the dataset. $AP_{i}$ is the average accuracy of the *i*-th sample, which indicates the accuracy of the prediction result for the *i*-th sample.

## 3 Experimental results and discussion

### 3.1 Comparison of the proposed method

In order to evaluate the counting performance, we use the test set of three sets of data, GWHD, WEDD and ESD datasets for testing. The specific results are shown in the **Table 2**. We show the improved model with Mask R-CNN (He et al., 2017), the original YOLACT (Bolya et al., 2019), YOLO v5, and two methods Blend Mask (Gao et al., 2022) and Cacade Mask (Zhang et al., 2022). And three evaluation metrics are used to evaluate our model. RMSE is a measure of the error between the predicted value and the ground-truth, which indicates the mean of the prediction error of the model and is more sensitive to large errors, so the smaller the metric is, the more it indicates that there are no certain images in our test data that have too large errors. Due to the introduction of density estimation to optimize detection results, our model has the best results in all three test sets, indicating that the improved model has better generalization ability. Density estimation was previously commonly used for crowd counting, and applying this method to wheat ear counting means that we have transformed the technology originally applicable to crowds into a tool to solve the wheat ear counting problem, further verifying the effectiveness of this method. In the three datasets, GWHD has the best performance with an RMSE of 1.29. Although Mask R-CNN and Cascade Mask R-CNN-based (Zhang et al., 2022) also have good performance, our model has a considerable advantage in terms of prediction speed. Bias is the systematic difference between the predicted value and the ground-truth. It represents the systematic discrepancy, whether the model consistently overestimates or underestimates the target variable. Minimizing bias is essential for improving the accuracy of predictions, and achieving a value closer to zero is indicative of better performance. Our model still achieves the best performance, with results of 0.8, 0.8, and 0.9 in the three datasets. This indicates that the overall results predicted by our model are very close to ground-truth, with no over or under-counting. The R² measures the linear relationship between predicted and ground-truth, and the closer it is to 1 indicating that the model is able to account for the greater amount of dependent variable variability, and the residuals account for the greater amount of total variability. Our model performs well, with the best result reaching R² of 0.9798 on the GWHD dataset.

**Table 2:** Comparison of counting results for different methods on multiple datasets.

Methods	GWHD			WEDD			ESD			FPS
	RMSE	Bias	R^²^	RMSE	Bias	R^²^	RMSE	Bias	R^²^	
Mask R-CNN (He et al., 2017)	1.52	1.2	0.9601	1.56	-0.9	0.9596	1.98	1.1	0.9541	7.96
BlendMask (Gao et al., 2022)	1.95	-1.3	0.9486	1.92	1.3	0.9432	1.89	1.2	0.9489	10.76
Cacade Mask (Zhang et al., 2022)	1.48	1.0	0.9523	1.53	-0.9	0.9541	1.61	-1.1	0.9511	6.54
YOLACT (Bolya et al., 2019)	1.83	1.2	0.9516	1.92	1.0	0.9547	2.49	-1.6	0.9416	13.52
YOLO v5	2.49	1.5	0.9364	2.12	1.5	0.9475	2.01	1.5	0.9517	20.51
Ours	1.29	0.8	0.9798	1.35	0.8	0.9716	1.45	0.9	0.9689	12.15

#### 3.1.1 Evaluation of segmentation performance

We used the same test images to evaluate the segmentation accuracy of the model using the evaluation metrics from the COCO dataset. The mAP denotes the average performance of the model under multiple IoU thresholds, and mAP50 and mAP75 are denoted as the values of mAP under thresholds of 0.50 a 0.75, respectively. The results are shown in **Table 3**. From the results, our model performs very well in terms of segmentation accuracy.

**Table 3:** Comparison of instance segmentation results for various approaches on multiple datasets.

Methods	GWHD			vWEDD			ESD		
	mAP	mAP50	mAP75	mAP	mAP50	mAP75	mAP	mAP50	mAP75
Mask R-CNN (He et al., 2017)	63.49	90.3ss5	79.16	60.35	88.76	78.96	62.29	90.62	80.34
BlendMask (Gao et al., 2022)	62.35	88.61	78.36	60.76	87.63	77.61	63.65	88.05	77.05
Cacade Mask (Zhang et al., 2022)	62.64	88.74	79.47	60.41	86.46	77.05	61.43	87.65	77.26
YOLACT (Bolya et al., 2019)	63.43	89.96	80.81	60.74	85.61	77.49	62.15	88.09	80.32
Ours	64.41	90.45	81.14	61.62	89.96	80.35	65.31	91.32	82.35

#### 3.1.2 Visual analysis of segmentation methods

**Figure 5:**
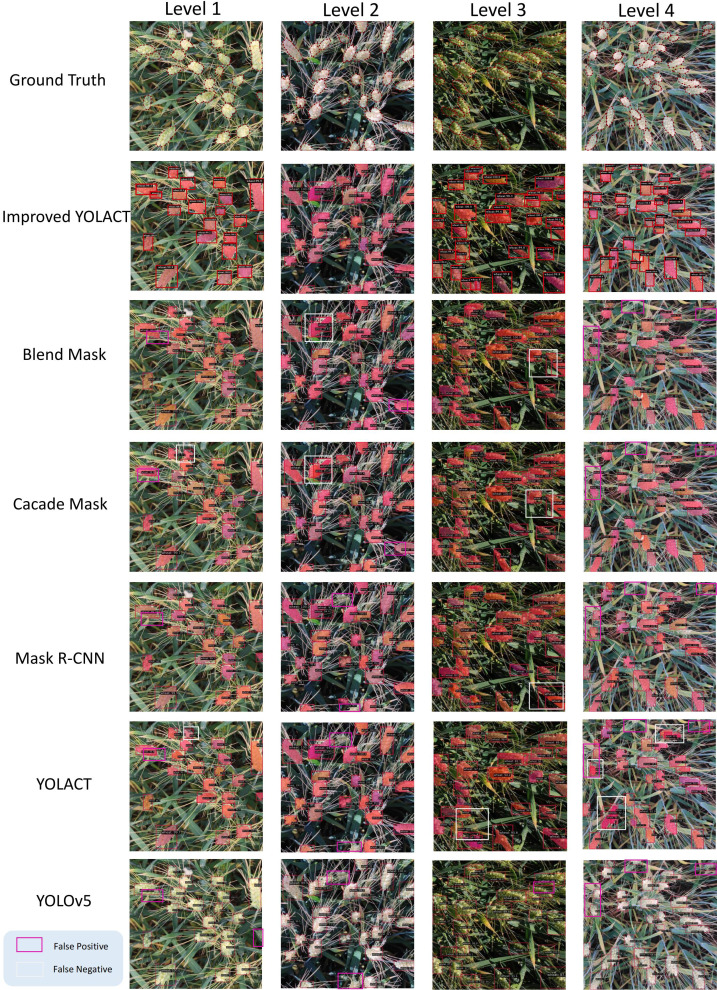
Test results for the self-constructed dataset, where blue boxes indicate False Positive and yellow boxes indicate False Negative. Levels 1 to 4 have increasing density.

**Figure 6:**
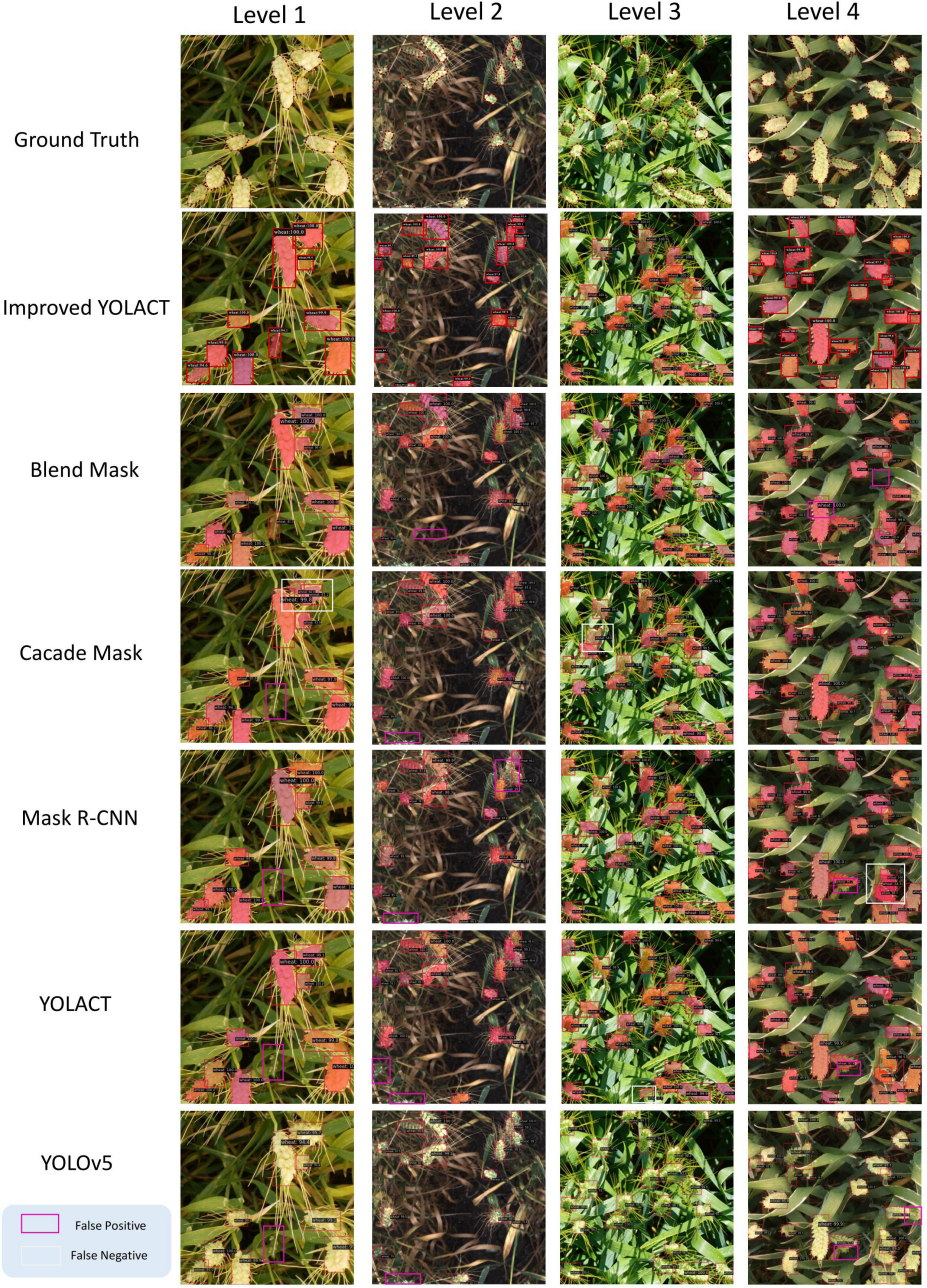
Test results for the WEDD dataset, where blue boxes indicate False Positive and yellow boxes indicate False Negative. Levels 1 to 4 have increasing density.

**Figure 7:**
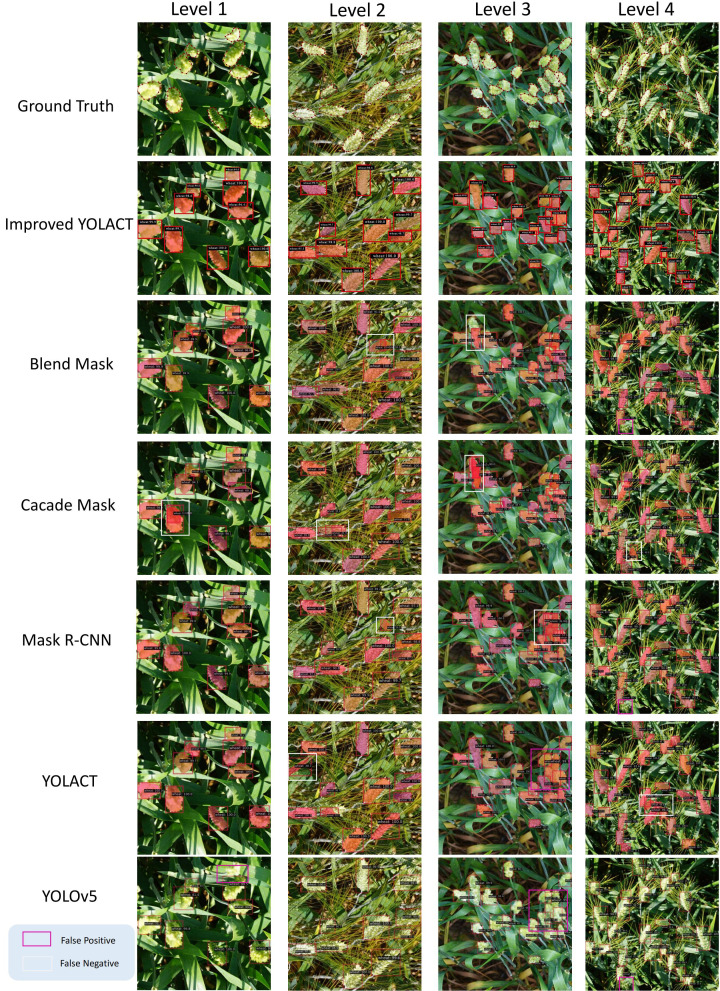
Test results for the GWHD dataset, where blue boxes indicate False Positive and yellow boxes indicate False Negative. Levels 1 to 4 have increasing density.

We tested each dataset individually and categorized the test images into four Levels based on different degrees of density, and the results for each dataset are shown as **Figures 5**–**7**, respectively. It is clear from these images that Level 1 and Level 2 shows that our method performs better relative to the other methods when the target density is low but there are some targets that are similar to the background (these targets may have similar color or morphology to the background, and sometimes wheat leaves in the background may be incorrectly identified as ears of wheat) or when the targets are occluded by wheat leaves. Due to our improved CBAM attention module, GeM pooling is resistant to noise and outliers. It uses a power-of-mean calculation, where outliers have less impact on the model, and pools multiple feature maps into a single representation when performing feature fusion. In contrast, other methods suffer from some false detection or omission problems in these cases.

In Level 3 through Level 4 results, when the targets are very dense, our method performs better due to the density map loss mechanism we introduced in training. In dense scenes, density map loss allows the model to better capture the distribution of targets. This helps to reduce the number of missed and false detections, thus improving the detection performance. From the results, the introduced density map branch has a significant advantage in dense target detection. At the same time, the improved CBAM attention module also provides better performance in cases where the target is similar to the background or is occluded. The combination of these two aspects enables our method to perform well in scenes with different densities.

### 3.2 Ablation study

In order to validate the effectiveness of our proposed model improvement in the wheat ears segmentation and counting task, we performed the validation on three different datasets: the GWHD, the WEDD, and Experimental Station Dataset(ESD), which cover different kinds of wheat ears images, including different lighting, viewing angles, and occlusion situations.

For the overall performance evaluation of segmentation, we also used the mAP metric, which can synthesize the performance of the model under different confidence thresholds **Table 4**. The experimental results show that YOLACT has the best mAP performance with the addition of Improved CBAM as well as the addition of density branches, and its mAP value is significantly higher than the other combinations. The two metrics, mAP50 and mAP75, which represent the segmentation performance at confidence thresholds of 0.5 and 0.75, respectively. The results show that our model also achieves the best performance in the three datasets. This indicates that the segmentation accuracy of the model is significantly improved after the introduction of the improved attention module and density module.

**Table 4:** Ablation experiment of segmentation metrics.

CBAM	Improved CBAM	Density	GWHD			WEDD			ESD		
			mAP	mAP50	mAP75	mAP	mAP50	mAP75	mAP	mAP50	mAP75
			63.43	89.96	80.81	60.74	85.61	77.49	62.15	88.09	80.32
✓			63.71	90.08	81.02	60.95	86.15	78.12	62.96	90.35	80.94
	✓		63.98	90.12	81.04	61.01	86.59	78.53	63.16	90.56	81.13
✓		✓	64.04	90.39	81.10	61.23	88.86	79.52	64.85	91.21	81.56
	✓	✓	64.41	90.45	81.14	61.62	89.96	80.35	65.31	91.32	82.35

In the counting task, we compared the performance of different models on wheat counting using RMSE, Bias and R² as certified evaluation metrics. The results are shown in **Table 5**. The results show that YOLACT+Improved CBAM+Density has the smallest RMSE value, the smallest Bias value and the highest R² value on all datasets, which improves the counting accuracy. Especially, the counting results are equally stable and robust at high densities. This implies that by incorporating an improved attention module into the model backbone and introducing a density branch after the FPN can achieve better overall performance on the ear segmentation and counting tasks.

**Table 5:** Ablation experiment of counting metrics.

CBAM	Improved CBAM	Density	GWHD			WEDD			ESD			FPS
			RMSE	Bias	R^²^	RMSE	Bias	R^²^	RMSE	Bias	R^²^	
			1.83	1.2	0.9516	1.92	1.0	0.9547	2.49	1.6	0.9416	13.52
✓			1.75	1.1	0.9595	1.83	1.0	0.9609	2.21	1.5	0.9549	13.21
	✓		1.57	1.0	0.9615	1.65	0.9	0.9653	2.05	1.4	0.9592	13.25
✓		✓	1.35	0.9	0.9686	1.43	0.9	0.9681	1.98	1.2	0.9612	12.34
	✓	✓	1.29	0.8	0.9798	1.35	0.8	0.9716	1.45	0.9	0.9689	12.15

The validation results on the three datasets consistently show that our proposed model improvement scheme achieves significant performance improvement on the wheat ears counting task. By introducing the improved attention module and density branching, we are able to better capture instances of wheat ears in the image and count the ears more accurately.

### 3.3 Discussion

#### 3.3.1 The effectiveness of GeM in image processing

Generalized mean pooling (GeM) is a commonly used pooling technique in deep learning architectures, which stands out compared to traditional pooling methods such as max pooling or average pooling due to its adaptability and enhanced representation power. As shown in **Table 4**, without any attention mechanism added to the model, the mAP in the GWHD, WEDD, and ESD datasets is merely 63.43%, 60.74%, and 62.15%, respectively. Introducing the unimproved CBAM leads to an increase in mAP by 0.37%, 0.21%, and 0.81%. This improvement arises from CBAM allowing for broader feature coverage over the objects of interest, facilitating better extraction of crucial feature information from the images. Upon replacing all pooling layers with GeM pooling, the mAP sees further enhancement, reaching 63.98%, 61.01%, and 63.16%. Compared to the initial two models, the performance boost in mAP after transitioning to GeM is significant. This is attributable to GeM dynamically adjusting pooling behavior based on data features, effectively reducing feature map dimensionality while preserving essential spatial information. GeM exhibits robustness against overfitting and lower sensitivity to minor input variations, thus facilitating better adaptation to the feature distributions across different datasets and tasks.

#### 3.3.2 The effectiveness of density map mechanisms in data analysis

Inspired by crowd counting, density map mechanisms are typically used for crowd counting. Wheat ears, similar to crowds, exhibit high density and may suffer from occlusion or overlap. By introducing density map estimation, we aim to optimize detection performance.

In the original YOLACT model, there are two branches: one for object detection and the other for instance segmentation. The object detection branch outputs category, bounding box information, and k mask coefficients for each object. The instance segmentation branch outputs k prototypes (mask prototype images) for the current input image. For each object, the k mask coefficients are multiplied by the k prototypes, and the results are summed. Then, a sigmoid non-linear function is applied to generate the final masks, resulting in instance segmentation for the object. We integrate density map estimation into the model’s detection branch. For each target, we obtain the central target coordinates and perform Gaussian smoothing on the two-dimensional coordinates of wheat in the image, mapping them onto a density map. The density map is fused with the detection head according to the target distribution. The network is trained with loss on the density branch, and back propagation increases the accuracy of the density map. Finally, the density map is linearly combined with the original object detection branch, enhancing detection performance. Specifically, as shown in **Table 4**, after improving CBAM without adding the density mechanism, the RMSE on three datasets is 1.57, 1.65, and 2.05, respectively. However, after introducing the density mechanism, the RMSE decreases by 0.28, 0.3, and 0.5. This improvement is attributed to the density map providing more accurate target information, leading to more precise detection by the detection branch.

#### 3.3.3 Model effectiveness

Due to our improvements to the original YOLACT model, where we replaced all pooling layers with GeM pooling, we can better capture crucial information within feature maps by combining and generalizing global max-pooling and global average-pooling. This adaptation allows our model to accommodate different tasks, data distributions, and even enables application in temporal feature aggregation. By incorporating density map mechanisms into the model, it exhibits enhanced detection capabilities in dense scenes, showcasing superior performance when handling dense targets. Specifically, as shown in **Table 3**, the Mask R-CNN achieves mAP scores of 63.49%, 60.35%, and 62.29% on three datasets respectively, while the original YOLACT model achieves mAP scores of 63.43%, 60.74%, and 62.15%. In contrast, our model achieves mAP scores of 64.41%, 61.62%, and 65.31%, demonstrating performance improvements across all datasets and outperforming other models.

#### 3.3.4 The reasons for performance differences across different datasets

This paper integrates three datasets, including two publicly available datasets and one custom dataset. The performance differences across these datasets arise from various factors related to the datasets themselves, such as differences in the variety of crops harvested, harvesting methods, growing conditions, data distribution, data quality, and domain adaptation issues.

For instance, in the GWHD dataset, as presented in **Table 4**, the original YOLACT model achieves an mAP of 63.43%. After introducing GeM and density map mechanisms, the mAP increases to 64.41%. This improvement can be attributed to the dataset containing a diverse range of wheat images, covering different varieties, geographical locations, climate conditions, and growth stages of wheat plants. Our model demonstrates more precise detection of various wheat images, benefiting from GeM’s ability to capture critical local and global features, thereby enhancing the representation capability of the model. In the WEDD dataset, the original YOLACT model achieves an mAP of 60.74%, while our improved model achieves an mAP of 61.62%, resulting in an improvement of 0.88%. The difference is due to the characteristic of the dataset where the background and wheat ears share similar colors. The improved model applies the concept of density estimation for detection and counting, enabling accurate detection even in complex backgrounds and minor color variations. In actual wheat fields, wheat ears typically have similar colors to surrounding vegetation or soil. Therefore, training the dataset with similar background colors helps simulate real-world environments and improves the model’s generalization ability in practical applications. In the ESD dataset, the original model achieves an mAP of 62.15%, whereas the improved model’s mAP increases to 65.31%. This indicates the effectiveness of our improvements with CBAM and density map integration. Data collected at different time periods capture the changes and evolution of the same objects in the same area, enhancing the dataset’s timeliness and comprehensiveness.

## 4 Conclusion

The main problem of the dense wheat ears segmentation counting task is that the targets and weeds are often occluded from each other in complex backgrounds, resulting in some targets not being completely segmented. In order to solve the problem of dense wheat ears segmentation and counting in complex environmental background, this paper proposes a wheat ears segmentation model based on density graph. Through ablation experiments and performance evaluation, the following conclusions are drawn:

1) Replacing the pooling layer of the CBAM module with the GeM pooling layer further improves the segmentation performance of the model in the dense wheat ears segmentation and counting task. The GeM pooling layer can be automatically adjusted according to the size and density of the target area, so that the model can handle targets of different scales and densities without manually adjusting the pooling parameters. The improved attention mechanism reduces the RMSE from 1.75 to 1.57, compared to the original CBAM. This improvement facilitates a more focused analysis of the spatial distribution of features, leading to reduced information loss in the spatial arrangement of targets.
2) Based on the improved CBAM, the R^2^ increases from 0.9615 to 0.9798 through pixel-level density estimation, the density map mechanism accurately discerns overlapping count targets, which can provide more granular information. The results show that with these key modifications, the density map-based YOLACT model further improves the accuracy of segmentation counting of dense wheat ears.

## Data availability statement

The original contributions presented in the study are included in the article/supplementary material. Further inquiries can be directed to the corresponding author.

## Author contributions

GZ: Writing – original draft, Writing – review & editing. ZW: Writing – original draft, Writing – review & editing. BL: Data curation, Formal analysis, Methodology, Writing – review & editing. LG: Data curation, Writing – review & editing. WZ: Data curation, Writing – review & editing. WY: Funding acquisition, Project administration, Writing – review & editing.
